# Inhibition of BET Proteins during Adolescence Affects Prefrontal Cortical Development: Relevance to Schizophrenia

**DOI:** 10.3390/ijms22168710

**Published:** 2021-08-13

**Authors:** Wiktor Bilecki, Agnieszka Wawrzczak-Bargieła, Iwona Majcher-Maślanka, Magdalena Chmelova, Marzena Maćkowiak

**Affiliations:** Laboratory of Pharmacology and Brain Biostructure, Department of Pharmacology, Maj Institute of Pharmacology, Polish Academy of Sciences, Smętna Str. 12, 31-343 Kraków, Poland; bilecki@if-pan.krakow.pl (W.B.); bargiela@if-pan.krakow.pl (A.W.-B.); majcher@if-pan.krakow.pl (I.M.-M.); chmelova@if-pan.krakow.pl (M.C.)

**Keywords:** bromodomain, neurodevelopment, JQ1, learning and memory, cognition

## Abstract

Background: The present study investigated the role of proteins from the bromodomain and extra-terminal (BET) family in schizophrenia-like abnormalities in a neurodevelopmental model of schizophrenia induced by prenatal methylazoxymethanol (MAM) administration (MAM-E17). Methods: An inhibitor of BET proteins, JQ1, was administered during adolescence on postnatal days (P) 23–P29, and behavioural responses (sensorimotor gating, recognition memory) and prefrontal cortical (mPFC) function (long-term potentiation (LTP), molecular and proteomic analyses) studies were performed in adult males and females. Results: Deficits in sensorimotor gating and recognition memory were observed only in MAM-treated males. However, adolescent JQ1 treatment affected animals of both sexes in the control but not MAM-treated groups and reduced behavioural responses in both sexes. An electrophysiological study showed LTP impairments only in male MAM-treated animals, and JQ1 did not affect LTP in the mPFC. In contrast, MAM did not affect activity-dependent gene expression, but JQ1 altered gene expression in both sexes. A proteomic study revealed alterations in MAM-treated groups mainly in males, while JQ1 affected both sexes. Conclusions: MAM-induced schizophrenia-like abnormalities were observed only in males, while adolescent JQ1 treatment affected memory recognition and altered the molecular and proteomic landscape in the mPFC of both sexes. Thus, transient adolescent inhibition of the BET family might prompt permanent alterations in the mPFC.

## 1. Introduction

Schizophrenia is a neurodevelopmental disorder whose first symptoms are usually diagnosed in late adolescence or early adulthood [1]. However, structural and functional abnormalities observed in individuals with schizophrenia are considered the result of early-life developmental disruption, and the neuromaturational process during adolescence appears to be important for the clinical aspects of the illness (reviewed in [1,2]). The latter observation is supported by several findings indicating that environmental or pharmacological interventions during childhood or adolescence might alter the course of schizophrenia (reviewed in [3,4]). The apparent lack of consistently replicated major genetic defects and the increasing evidence of changes in gene expression after exposure to environmental risk factors suggest that epigenetic regulation might be involved in the aetiology of schizophrenia (reviewed in [5]). However, limited data related to epigenetic regulation, especially in adolescence, of alterations in the developmental trajectory of the brain predisposed genetically or environmentally to schizophrenia symptom appearance are available.

The histone acetylation pattern is one of the epigenetic modifications that might be critical to gene transcription and involved in the brain maturation process [6]. Moreover, previous data from our [7] and other [8] studies using animal neurodevelopmental models of schizophrenia indicated that the regulation of histone acetylation in the medial prefrontal cortex (mPFC) during early adolescence might be an important mechanism involved in the development of schizophrenia-like abnormalities in adulthood. The pattern of acetylation marks is recognised by acetyl-lysine residues such as bromodomain (epigenetic readers) functioning as a scaffold for assembly of macromolecular complexes that alter chromatin accessibility to transcription factors and allow the recruitment or activation of RNA polymerases [9]. These complexes then initiate transcriptional programmes that result in altered gene expression. Epigenetic readers of acetylation marks from the bromodomain and extra-terminal domain (BET) family have been shown to be key regulators of chromatin dynamics and the disease-associated acetylome [9,10]. The BET family is comprised of four proteins: BRD2, BRD3, and BRD4, which are expressed in most cells and tissues, and BRDT that is primarily localised in the testes [11]. Acetyl-lysine mimic compounds, namely, BET inhibitors (JQ1, I-BET, and MS417) with high affinity and specificity to BET bromodomains, function by blocking BET proteins from binding acetylated chromatin. Compounds regulating the function of BET proteins might alter epigenetic status and affect transcriptional activity in cells [9,10].

Some evidence indicates the involvement of BET proteins in neuroplasticity regulation and memory formation, as well as their potential roles in the pathomechanisms of neurodevelopmental disorders [12,13,14]. Findings from animal studies showed a selective upregulation of BRD4 protein in an animal model of Fragile X Syndrome (FXS), a neurodevelopmental disorder that might cause intellectual disability or autism spectrum disorder [13]. An inhibition of BRD4 function diminished transcriptional disruption and reversed behavioural deficits observed in an FXS animal model [13]. On the other hand, pharmacological suppression of BET protein during mice adolescence led to selective repression of neuronal gene expression and development of autism-like syndrome [15]. However, BET protein involvement in pathogenesis of other neurodevelopmental disorders, i.e., schizophrenia, remains unknown. Therefore, in the present study, we investigated the role of the BET protein family in early adolescence, the period of proper neurodevelopment and emergence of schizophrenia-like abnormalities, in a neurodevelopmental animal model of schizophrenia induced by prenatal administration of an antimitotic agent, methylazoxymethanol (MAM), at embryonic day 17 (MAM-E17 model) [16]. We determined whether the administration of the BET family inhibitor JQ1 during early adolescence (postnatal day (P) 23–P29) may affect the behavioural response in adult rats and mPFC neurodevelopment. Our previous study using the MAM-E17 model showed that the chosen period is a critical window for epigenetically driven mPFC maturation and the development of behavioural and neurochemical schizophrenia-like abnormalities in adult MAM-E17 rats [7,17,18]. Therefore, in the present study, we analysed behavioural responses related to cognitive function: sensorimotor gating [19,20] and recognition memory [21,22]. An electrophysiological study measuring long-term potentiation (LTP) was also performed to determine adult mPFC function at the cellular level. Moreover, the expression of genes in the BET family (*BRD2*, *BRD3*, and *BRD4*) was measured to verify an effect of adolescent BET protein inhibition on the function of the BET family in the adult mPFC. In addition, neuronal activation in the mPFC was examined by measuring the expression of immediate early genes (IEGs: *c-Fos*, activity-regulated cytoskeletal (*Arc*), and neuronal PAS domain protein 4 (*Npas4*)) [23] that might be regulated by BET proteins [12].

The cortical dysfunction of GABAergic transmission observed in the prefrontal cortex of individuals with schizophrenia might result from developmental abnormalities [24], which was also confirmed in our previous study showing impaired transcription of genes that are markers of the GABA system, such as glutamic acid decarboxylase (GAD)67 and parvalbumin (PV), in the adult mPFC of MAM-E17 animals [17,25]. Thus, we verified that the expression of *GAD67* and *PV* genes in the adult mPFC was related to dysfunction of BET proteins during adolescence. In addition to investigating the functional and molecular aspects of adolescent inhibition of BET proteins in the adult mPFC, we also designed a proteomic study to analyse the pattern of protein alterations in the adult mPFC induced by adolescent disruption of BET protein function.

The experiments were designed to assess both sexes (males and females) because of findings showing sex differences in psychiatric disorders, i.e., schizophrenia [26]. Moreover, in the MAM model of schizophrenia, different results related to the development of schizophrenia-like abnormalities are observed in males and females [27,28,29]. Thus, in our study, we also investigated whether adolescent inhibition of the BET family might exert a sex-dependent effect on the development of schizophrenia-like dysfunction.

## 2. Results

### 2.1. Behavioural Response 

We studied recognition memory and sensorimotor gating in both males and females to determine whether adolescent BET protein inhibition affected cognitive function in MAM-E17 rats.

#### 2.1.1. Recognition Memory in Males

**Figure 1:**
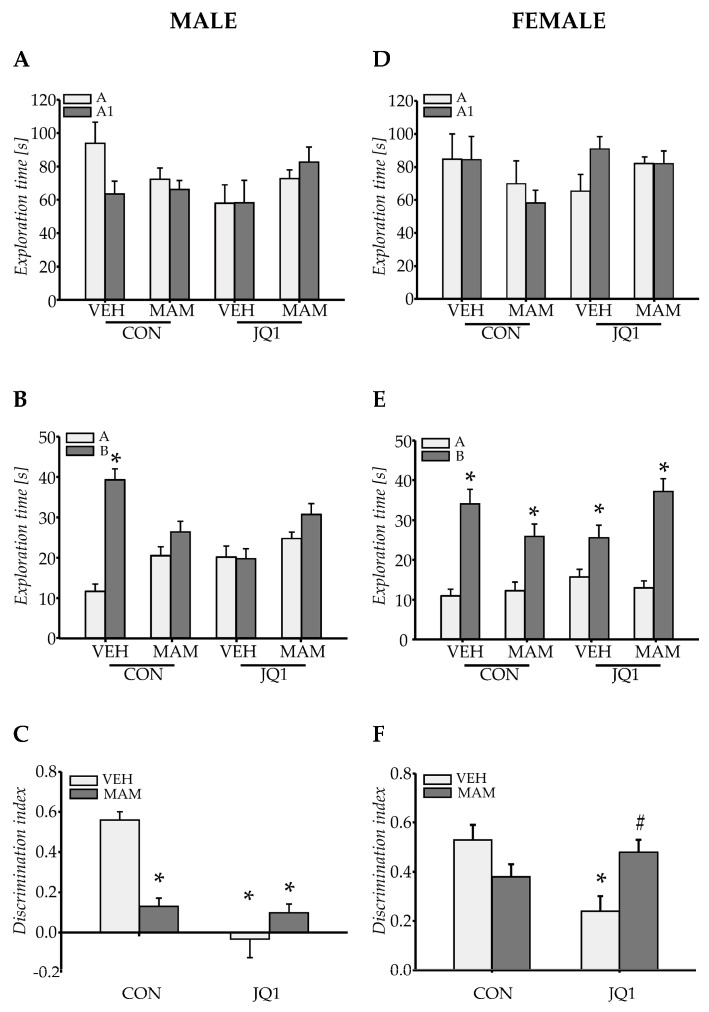
The effect of adolescent JQ1 administration on recognition memory in the novel object recognition test in the MAM-E17 model of schizophrenia. (**A**,**D**) Exploration time of two identical objects in the acquisition session, (**B**,**E**) exploration time of one novel and one familiar object in the retention test conducted 1 h following the acquisition session, (**C**,**F**) discrimination index. The rats were (males: **A**–**C** and females: **D**–**F**) exposed to JQ1 or vehicle (CON) in early adolescence (P23–P29), and the analyses were performed in rats at P60. Each data point represents the mean ± SEM; n = 12 per group. **B**—* *p* < 0.05 vs. familiar object (A), **C**—* *p* < 0.05 vs. VEH-CON, # *p* < 0.05 vs. MAM-CON (two-way repeated measures ANOVA (**A**,**B**,**D**,**E**) or two-way ANOVA (**C**,**F**) followed by a Tukey test).

MAM treatment did not induce any significant differences in the time spent exploring two identical objects presented during the training session (prenatal factor × object: F (1, 44) = 3.78, *p* = 0.06), and no significant differences were observed between groups (prenatal × postnatal factors × object: F (1, 44) = 0.27; *p* = 0.61; Figure 1A).

However, in the retention session, MAM exerted statistically significant effects on discrimination of the novel object from the familiar object (prenatal factor × object: F (1, 44) = 14.93; *p* < 0.0004), and an interaction between MAM administration and adolescent JQ1 treatment was observed (prenatal × postnatal factors × object: F (1, 44) = 51.13; *p* < 0.000001). VEH-CON rats spent significantly more time exploring the novel object than the familiar object (*p* < 0.003, Figure 1B). However, MAM-CON and MAM-JQ1 rats did not discriminate between the objects and spent similar amounts of time exploring each object (*p* = 0.065, Figure 1B). Adolescent JQ1 administration affected the exploration of the novel object by rats in the VEH group, and these animals did not discriminate between the objects and spent similar amounts of time exploring each object (*p* = 0.99, Figure 1B).

A significant effect of MAM treatment on the discrimination index was observed (prenatal factor: F (1, 44) = 7.7; *p* < 0.009), and a significant interaction between MAM and JQ1 was identified (prenatal × postnatal factors: F (1, 44) = 27.44; *p* < 0.000005). A similar decrease in the discrimination index was observed in all groups when compared to the VEH-CON group (*p* < 0.0002); however, the strongest effect was observed in the VEH-JQ1 group (Figure 1C).

#### 2.1.2. Recognition Memory in Females

MAM treatment did not induce any significant differences in time spent exploring two identical objects presented during the training session (prenatal factor × object: F (1, 44) = 0.73; *p* = 0.4), and significant differences were not observed between groups (prenatal × postnatal factors × object: F (1, 44) = 0.14; *p* = 0.71; Figure 1D).

In the retention session, MAM did not affect the discrimination of the novel object from the familiar object (prenatal factor × object: F (1, 44) = 0.72; *p* = 0.4); however, an interaction between MAM administration and adolescent JQ1 treatment was observed (prenatal × postnatal factors × object: F (1, 44) = 17.23; *p* < 0.0002). All groups spent significantly more time exploring the novel object than the familiar object (*p* < 0.0002 for the VEH-CON group, *p* < 0.0006 for the MAM-CON group, *p* < 0.02 for the VEH-JQ1 group, and *p* < 0.0002 for the MAM-JQ1 group; Figure 1E).

MAM also did not alter the discrimination index (prenatal factor: F (1, 44) = 0.68; *p* = 0.41); however, an interaction between MAM administration and adolescent JQ1 treatment was observed (prenatal × postnatal factors: F (1, 44) = 14.69; *p* < 0.0005). MAM alone did not change the discrimination index (*p* = 0.18 compared with the VEH-CON group). However, JQ1 decreased the discrimination index in the VEH group (*p* < 0.002 compared with the VEH-CON group and *p* < 0.02 compared with the MAM-JQ1 group), and that effect was not observed in the MAM-JQ1 group (*p* = 0.91 compared with the VEH-CON group, Figure 1F).

The results showed a deficit in recognition memory only in the female VEH-JQ1 group.

#### 2.1.3. Sensorimotor Gating in Males 

**Figure 2:**
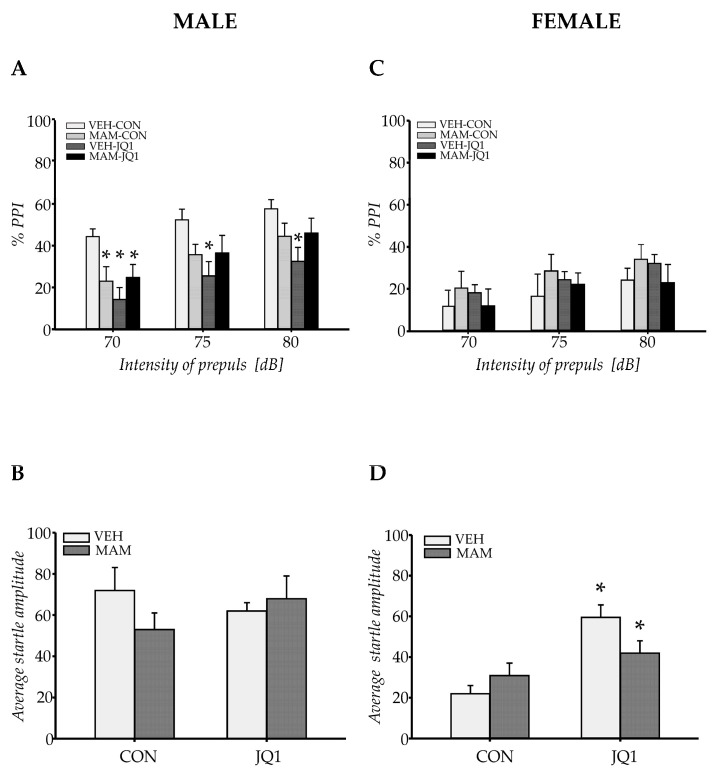
The effects of an adolescent JQ1 administration on sensorimotor gating (% of pre-pulse inhibition, PPI) in the acoustic startle response test (**A**,**C**) and on the average amplitude of startle (**B**,**D**) in the MAM-E17 model of schizophrenia. The rats (males: **A**,**B** and females: **C**,**D**) were exposed to JQ1 or vehicle (CON) in early adolescence (P23–P29), and the analyses were performed in rats at P70. Each data point represents the mean ± SEM; n = 12 per group * *p* < 0.05 vs. VEH-CON (two-way repeated measures ANOVA (**A**,**C**) or two-way ANOVA (**B**,**D**) followed by a Tukey test).

The acoustic pre-pulse intensity exerted significant effects on PPI (F (2, 88) = 36.76, *p* < 0.0000001, Figure 2A).

The results revealed that either MAM or JQ1 administration affected recognition memory in males.

MAM treatment did not affect PPI in the adult rats (prenatal factor: F (1, 44) = 0.197; *p* = 0.66), and the effect of MAM was not determined by the pre-pulse intensity used (prenatal factor × pre-pulse: F (2, 88) = 0.61; *p* = 0.54). However, adolescent JQ1 administration altered PPI in adulthood (postnatal factor: F (1, 44) = 5.76; *p* < 0.03), but the effect of JQ1 was not determined by the pre-pulse intensity used (postnatal factor × pre-pulse: F (2, 88) = 0.058; *p* = 0.95). Moreover, a statistically significant interaction between MAM and JQ1 treatment was observed (prenatal × postnatal factors: F (1, 44) = 7.72; *p* < 0.009); however, the effect did not depend on the intensity of the applied pre-pulse (prenatal × postnatal factors × pre-pulse: F (2, 88) = 0.44, *p* = 0.65, Figure 2A).

MAM induced a statistically significant decrease in PPI only at the lowest pre-pulse intensity used (*p* < 0.04 at 70 dB compared with the VEH-CON group). JQ1 administration decreased PPI in the VEH group at all applied pre-pulse intensities (*p* < 0.03 at 70 dB, *p* < 0.05 at 75 dB, and *p* < 0.005 at 80 dB for the comparison of the VEH-CON group with the appropriate group); however, JQ1 did not alter PPI in the MAM-treated group (Figure 2A).

In addition, the average startle amplitude was analysed in each experiment. Neither MAM nor JQ1 altered the average startle amplitude (prenatal × postnatal factors: F (1, 44) = 5.24; *p* = 0.14, Figure 2B).

The results revealed a deficit in sensorimotor gating in all analysed male groups, however the strongest effect was observed in the VEH-JQ1 group.

#### 2.1.4. Sensorimotor Gating in Females 

Significant effects of the acoustic pre-pulse intensity on PPI were observed F (2, 88) = 18.8, *p* < 0.0000001, Figure 2C).

Neither MAM (prenatal factor × pre-pulse: F (2, 88) = 0.62; *p* = 0.54) nor adolescent JQ1 treatment (postnatal factor × pre-pulse: F (2, 88) = 0.16; *p* = 0.85) affected PPI at all applied pre-pulse intensities. An interaction between factors was not observed (prenatal × postnatal factors × pre-pulse: F (2, 88) = 0.16; *p* = 0.86, Figure 2C).

However, the average startle amplitude analysed in each experiment was affected by adolescent JQ1 treatment (postnatal factor: F (1, 44) 21.53; *p* < 0.00004). MAM did not alter the average startle amplitude (prenatal factor: F (1, 44) = 0.68; *p* = 0.42); however, an interaction between MAM and JQ1 treatment was observed (prenatal × postnatal factors: F (1, 44) = 6.62; *p* < 0.014). Adolescent administration of JQ1 increased the startle amplitude in both the VEH- and MAM-treated groups (*p* < 0.0003 and *p* < 0.05 respectively, compared with the VEH-CON group, Figure 2D).

Neither MAM nor JQ1 administration affected sensorimotor gating in females, however adolescent JQ1 treatment increased the average startle amplitude in both the VEH- and MAM-treated groups.

### 2.2. Electrophysiological Recordings in the Adult Medial Prefrontal Cortex

We studied long-term potentiation in both males and females to investigate whether prenatal MAM administration and/or adolescent exposure to JQ1 affects functional synaptic plasticity in the mPFC.

#### 2.2.1. LTP in Males

Significant effects of tetanisation were observed (tetanisation: F (1, 112) = 62.98, *p* < 0.000001). Both prenatal and postnatal treatment factors affected tetanisation of the field potential amplitude (prenatal factor × tetanisation: F (1, 112) = 14.29; *p* < 0.0003) and postnatal factor × tetanisation: F (1, 112) = 5.51; *p* < 0.03). No statistically significant interactions between the analysed factors and tetanisation were observed (prenatal × postnatal factors × tetanisation: F (1, 112) = 0.25, *p* = 0.62)).

**Figure 3:**
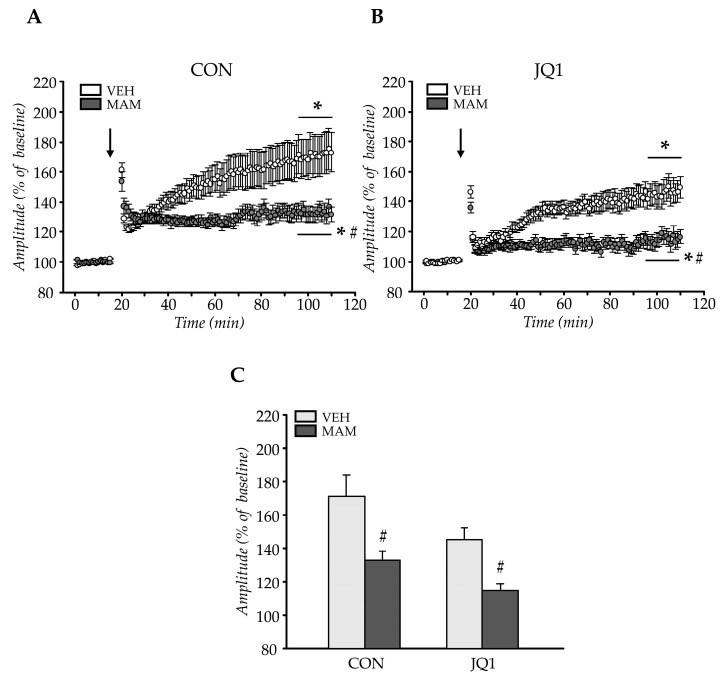
The effects of an adolescent JQ1 administration on long-term potentiation (LTP) in the male adult medial prefrontal cortex in the MAM-E17 model of schizophrenia. The rats were exposed to JQ1 or vehicle (CON) in early adolescence (P23–P29), and the analyses were performed in rats at P60–P110. The data are presented as group mean ± SEM, n = 12–17 (the number of brain sections from 6 to 9 animals). The data are expressed as percentage of the baseline amplitude values for each time point (**A**–**C**), **C**—the average of LTP during the last 15 min of recording. * *p* < 0.05 compared with the last 15 min of pre-tetanisation recording (for values averaged over the last 15 min of recording); # *p* < 0.05 compared with VEH-CON animals (for values averaged over the last 15 min of recording); two-way repeated measures ANOVA (**A**,**B**) or two-way ANOVA (**C**) followed by a Tukey test. Arrows indicate time of delivery of tetanic stimulation.

An analysis of the magnitude of potentiation revealed that the MAM groups generally displayed a smaller potentiation than the VEH-CON rats (*p* < 0.006 for the MAM-CON group and *p* < 0.00005 for the MAM-JQ1 group). However, no differences in LTP were observed in VEH-JQ1 rats (*p* = 0.24 compared with the VEH-CON group, Figure 3).

The results showed a deficit in LTP in the adult mPFC of the MAM-treated males, and JQ1 treatment did not affect LTP formation in any analysed group.

#### 2.2.2. LTP in Females

**Figure 4:**
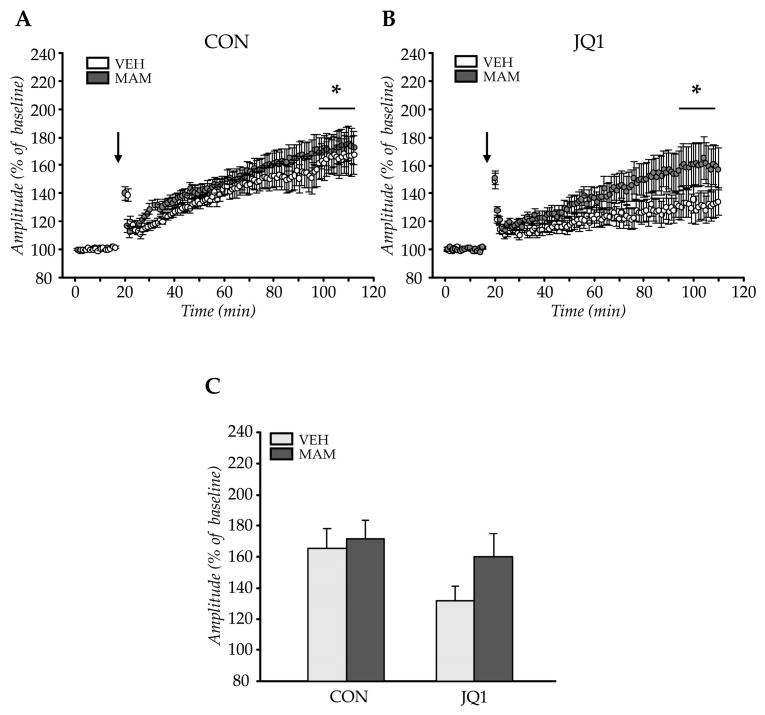
The effects of an adolescent JQ1 administration on long-term potentiation (LTP) in the female adult medial prefrontal cortex in the MAM-E17 model of schizophrenia. The rats were exposed to JQ1 in early adolescence (P23–P29) or vehicle (CON), and the analyses were performed in rats at P60–P110. The data are presented as group mean ± SEM, n = 12–17 (the number of brain sections from 6 to 9 animals). The data are expressed as percentage of the baseline amplitude values for each time point (**A**–**C**), **C**—the average of LTP during the last 15 min of recording. * *p* < 0.05 compared with the last 15 min of pre-tetanisation recording (for values averaged over the last 15 min of recording); two-way repeated measures ANOVA (**A**,**B**) or two-way ANOVA (**C**) followed by a Tukey test. Arrows indicate time of delivery of tetanic stimulation.

Significant effects of tetanisation were observed (tetanisation: F (1, 115) = 69.32; *p* < 0.000001). Neither prenatal nor postnatal treatment factors affected tetanisation of the field potential amplitude (prenatal factor × tetanisation: F (1,115) = 0.81; *p* = 0.37 and postnatal factor × tetanisation: F (1, 115) = 2.19; *p* = 0.14). Statistically significant interactions between the analysed factors and tetanisation were not observed (F (1,115) = 0.26, *p* = 0.61, Figure 4).

Neither prenatal MAM nor postnatal JQ1 administration affected LTP formation in the female adult mPFC.

### 2.3. Molecular Studies in the Adult Medial Prefrontal Cortex

#### 2.3.1. BET Family Genes

We analysed the mRNA levels of *BRD2*, *BRD3*, and *BRD4* in the mPFC of both males and females to determine whether adolescent JQ1 administration affected the transcription of the BET family.

- *BRD2* expression

**Figure 5:**
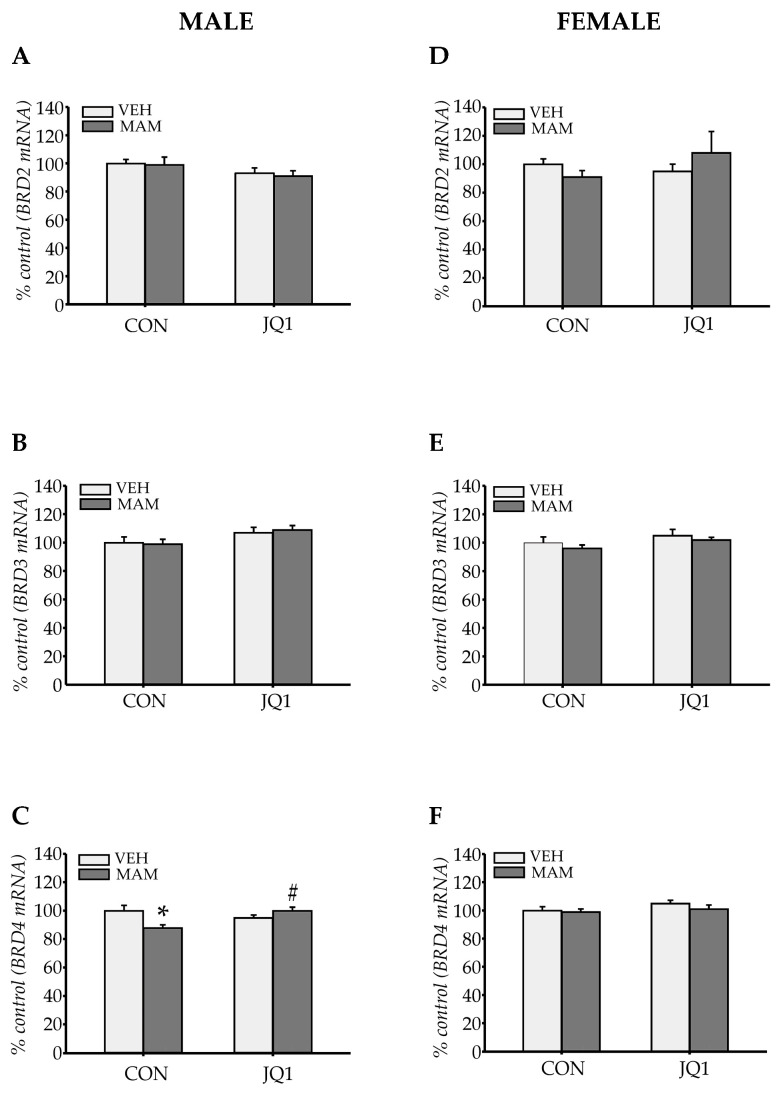
The effects of an adolescent JQ1 administration on *BRD* gene expression in the adult medial prefrontal cortex in the MAM-E17 model of schizophrenia. The rats (males: **A**–**C** and females: **D**–**F**) were exposed to JQ1 or vehicle (CON) in early adolescence (P23–P29), and the analyses were performed in rats at P110. (**A**–**C**) *BRD2*, *BRD3*, and *BRD4* expression in males, respectively. (**D**–**F**) *BRD2*, *BRD3*, and *BRD4* expression in females, respectively. The data are expressed as percentage of VEH-CON. Each data point represents the mean ± SEM; n = 6 per group; * *p* < 0.05 vs. VEH-CON, # *p* < 0.05 vs. MAM-CON (two-way ANOVA followed by a Tukey test).

Prenatal MAM administration did not alter the expression of the *BRD2* mRNA in the mPFC of males (prenatal factor: F (1, 20) = 0.12; *p* = 0.73). No interaction was observed between the two analysed factors (prenatal × postnatal factors: F (1, 20) = 0.001; *p* = 0.98, Figure 5A).

The lack of an effect of MAM on *BRD2* expression was also observed in the female mPFC (prenatal factor: F (1, 20) = 0.04; *p* = 0.84), and no interaction was observed between the two analysed factors (prenatal × postnatal factors: F (1, 20) = 1.28; *p* = 0.27, Figure 5D).

- *BRD3* expression

Prenatal MAM administration did not alter the expression of the *BRD3* mRNA in the mPFC of males (prenatal factor: F (1, 20) = 0.04; *p* = 0.84), but an effect of adolescent JQ1 treatment was observed (postnatal factor: F (1, 20) = 5.94; *p* < 0.03). However, a statistically significant interaction between MAM and JQ1 treatment was not found (prenatal × postnatal factors: F (1, 20) = 0.17; *p* = 0.69, Figure 5B).

Prenatal MAM administration or an interaction between analysed factors did not alter *BRD3* expression in the female mPFC (prenatal factor: F (1, 20) = 1.53; *p* = 0.23 and prenatal × postnatal factors: F (1, 20) = 0.017; *p* = 0.89, Figure 5E).

- *BRD4* expression

Neither of the analysed factors affected *BRD4* expression in the male mPFC (prenatal factor: F (1, 20) = 2.1; *p* = 0.16, postnatal factor: F (1, 20) = 1.64; *p* = 0.21). However, a statistically significant interaction between factors was identified (prenatal × postnatal factors: F (1, 20) = 12.86; *p* < 0.002). A decrease in *BRD4* mRNA levels was observed in the MAM-CON group (*p* < 0.001 compared with the VEH-CON group), and this effect was abolished by adolescent JQ1 administration (*p* = 0.99 compared with the VEH-CON group and *p* < 0.02 compared with the MAM-CON group, Figure 5C).

In contrast, neither MAM nor JQ1 affected *BRD4* mRNA levels in the female mPFC (prenatal factor: F (1, 20) = 1.22; *p* = 0.28, postnatal factor: F (1, 20) = 2.78; *p* = 0.11). An interaction between analysed factors was not observed (prenatal × postnatal factors: F (1, 20) = 0.4; *p* = 0.53, Figure 5F).

Thus, prenatal MAM administration decreased *BRD4* expression only in males, and this effect was reversed by adolescent JQ1 treatment. However, neither MAM nor JQ1 administration affected *BRD2* and *BRD3* expression in the adult mPFC of either sex.

#### 2.3.2. Immediate Early Genes

We analysed levels of the *c-Fos*, *Npas4*, and *Arc* mRNAs as markers of cellular activity in the mPFC of both males and females to determine whether adolescent JQ1 administration affected neuronal and synaptic activity at the molecular level.

- *c-Fos* expression

**Figure 6:**
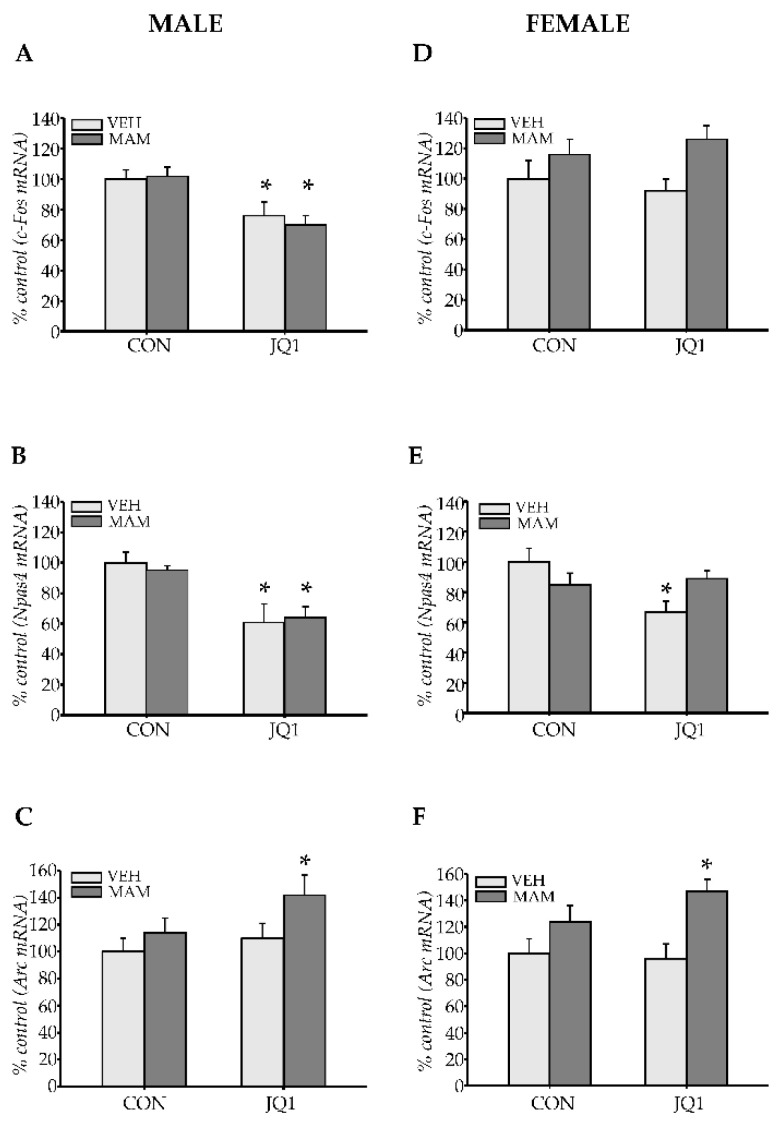
The effects of an adolescent JQ1 administration on early gene expression (*c-Fos*, *Arc*, *Npas4*) in the adult medial prefrontal cortex in the MAM-E17 model of schizophrenia. The rats (males: **A**–**C** and females: **D**–**F**) were exposed to JQ1 or vehicle (CON) in early adolescence (P23–P29), and the analyses were performed in rats at P110. (**A**–**C**) *c-Fos*, *Arc*, and *Npas4* expression in males, respectively. (**D**–**F**) *c-Fos*, *Arc*, and *Npas4* expression in females, respectively. The data are expressed as percentage of VEH-CON. Each data point represents the mean ± SEM; n = 6 per group * *p* < 0.05 vs. VEH-CON (two-way ANOVA followed by a Tukey test).

Prenatal MAM administration did not affect *c-Fos* expression in the male mPFC (prenatal factor: F (1, 20) = 0.0014; *p* = 0.97); however, an effect of JQ1 treatment was observed (postnatal factor: F (1, 20) = 0.57; *p* < 0.0002). An interaction between factors was not noted (prenatal × postnatal factors: F (1, 20) = 0.13; *p* = 0.72). A decrease in *c-Fos* mRNA levels was observed in the VEH-JQ1 and MAM-JQ1 groups (*p* < 0.03 and *p* < 0.02 compared with the VEH-CON group respectively, Figure 6A).

In contrast, MAM altered *c-Fos* expression in the female mPFC (prenatal factor: F (1, 20) = 7.45; *p* < 0.02); however, JQ1 did not exert any effect (postnatal factor: F (1, 20) = 0.01; *p* = 0.91). An interaction between analysed factors was not observed (prenatal × postnatal factors: F (1, 20) = 0.93; *p* = 0.35), and a difference in mRNA levels between groups was not detected (Figure 6D).

- *Npas4* expression

MAM administration did not alter *Npas4* expression in the male mPFC (prenatal factor: F (1, 20) = 0.02; *p* = 0.88); however, JQ1 treatment exerted a significant effect (postnatal factor: F (1, 20) = 22.4; *p* < 0.0002). An interaction between factors was not observed (prenatal × postnatal factors: F (1, 20) = 0.29; *p* = 0.59). A significant decrease in the level of the *Npas4* mRNA was observed in the VEH-JQ1 and MAM-JQ1 groups (*p* < 0.007 and *p* < 0.02 compared with the VEH-CON group respectively, Figure 6B).

MAM treatment also did not alter *Npas4* expression in the female mPFC (prenatal factor: F (1, 20) = 0.24; *p* = 0.63), and JQ1 treatment exerted a significant effect (postnatal factor: F (1, 20) = 4.4; *p* < 0.05). Moreover, an interaction between factors was observed (prenatal × postnatal factors: F (1, 20) = 7.73; *p* < 0.02). A significant decrease in the level of the *Npas4* mRNA was observed only in the VEH-JQ1 group (*p* < 0.02 compared with the VEH-CON group, Figure 6E).

- *Arc* expression

MAM administration altered *Arc* expression in the male mPFC (prenatal factor: F (1, 20) = 5.03; *p* < 0.04). However, JQ1 treatment did not modulate the level of the *Arc* mRNA (postnatal factor: F (1, 20) = 3.22; *p* = 0.088), and no interaction was observed between the analysed factors (prenatal × postnatal factors: F (1, 20) = 0.69; *p* = 0.41). A significant increase in the *Arc* mRNA level was detected in the MAM-JQ1 group (*p* < 0.05 compared with the VEH-CON group, Figure 6C).

Similar alterations were observed in females, and MAM treatment altered *Arc* expression in the female mPFC (prenatal factor: F (1, 20) = 13.37; *p* < 0.002). JQ1 did not change the level of the *Arc* mRNA (postnatal factor: F (1, 20) = 0.86; *p* = 0.36), and no interaction was observed between the analysed factors (prenatal × postnatal factors: F (1, 20) = 1.67; *p* = 0.21). A significant increase in *Arc* mRNA levels was detected in the MAM-JQ1 group (*p* < 0.02 compared with the VEH-CON group, Figure 6F).

Thus, prenatal MAM administration did not alter expression of the analysed immediate early genes in the adult mPFC of both sexes. However, JQ1 treatment repressed transcription of *c-Fos* and *Npas4* genes in both the VEH and MAM groups of males, but in females, the *Npas4* mRNA level was decreased only in the VEH group. In contrast, JQ1 administration increased *Arc* expression in the MAM-treated groups of both sexes.

#### 2.3.3. GABAergic Markers

The expression of the *GAD67* and *PV* mRNAs was examined to determine whether adolescent inhibition of BET proteins affected GABAergic interneurons.

- *GAD67* expression

**Figure 7:**
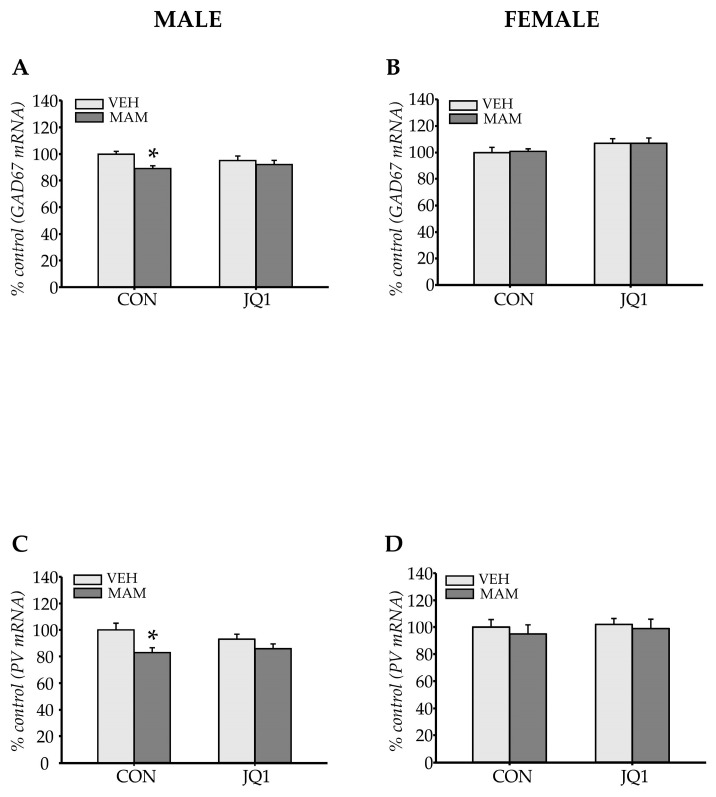
The effects of an adolescent JQ1 administration on *GAD67* and *parvalbumin (PV)* gene expression in the adult medial prefrontal cortex in the MAM-E17 model of schizophrenia. The rats (males and females) were exposed to JQ1 or vehicle (CON) in early adolescence (P23–P29), and the analyses were performed in rats at P110. (**A**,**C**) *GAD67* and *PV* expression in males, respectively. (**B**,**D**) *GAD67* and *PV* expression in females, respectively. The data are expressed as percentage of VEH-CON. Each data point represents the mean ± SEM; n = 6 per group * *p* < 0.05 vs. VEH-CON (two-way ANOVA followed by a Tukey test).

MAM administration altered *GAD67* expression in the male mPFC (prenatal factor: F (1, 20) = 7.14; *p* < 0.02). However, JQ1 treatment did not change the level of the *GAD67* mRNA (postnatal factor: F (1, 20) = 0.35; *p* = 0.56), and no interaction was observed between the analysed factors (prenatal × postnatal factors: F (1, 20) = 2.76; *p* = 0.11). A significant decrease in *GAD67* mRNA levels was detected in the MAM-CON group (*p* < 0.03 compared with the VEH-CON group, Figure 7A).

Prenatal MAM administration did not alter *GAD67* expression in the female mPFC (prenatal factor: F (1, 20) = 0.016; *p* = 0.90); however, an effect of JQ1 treatment was observed (postnatal factor: F (1, 20) = 4.55; *p* < 0.05). An interaction between factors was not noted (prenatal × postnatal factors: F (1, 20) = 0.072; *p* = 0.79), and no statistically significant differences in *GAD67* mRNA levels were observed among groups (Figure 7B).

- *PV* expression

MAM administration altered *PV* expression in the male mPFC (prenatal factor: F (1, 20) = 10.51; *p* < 0.005). However, JQ1 treatment did not change the level of the *PV* mRNA (postnatal factor: F (1, 20) = 0.33; *p* = 0.57), and no interaction was observed between the analysed factors (prenatal × postnatal factors: F (1, 20) = 1.86; *p* = 0.19). A significant decrease in *PV* mRNA levels was detected in the MAM-CON group (*p* < 0.02 compared with the VEH-CON group, Figure 7C).

Neither of the analysed factors affected *PV* expression in the female mPFC (prenatal factor: F (1, 20) = 0.45; *p* = 0.51, postnatal factors: F (1, 20) = 0.26; *p* = 0.62). An interaction between the analysed factors was not observed (prenatal × postnatal factors: F (1, 20) = 0.052; *p* = 0.82, Figure 7D).

Thus, the prenatal MAM factor decreased both *GAD67* and *PV* expression only in the male mPFC of the MAM-treated group. Postnatal JQ1 administration did not affect either *GAD67* or *PV* expression in either sex.

#### 2.3.4. Correlation Analysis of Gene Expression and Behavioural Response

**Figure 8:**
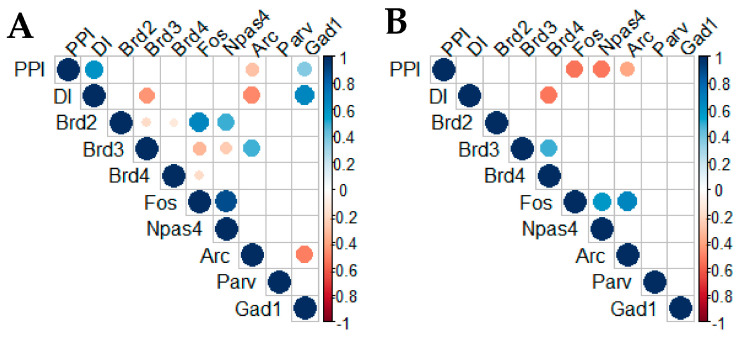
The matrix of correlation plots demonstrates correlations among the levels of the studied mRNA’s in the adult medial prefrontal cortex and behavioural data in males (**A**) and females (**B**) (Pearson’s correlation, *p* < 0.05). PPI—pre-pulse inhibition, DI—discrimination index. The colour code follows the indicated values of the correlation coefficient. The colour intensity and the size of the circles are proportional to the correlation coefficients. Two basic colours represent negative (red) or positive (blue) correlations.

The behavioural tests and gene expression analyses were completed on the same animals. Thus, a correlation analysis was performed to determine the associations between behavioural responses and changes in gene expression in the adult mPFC (Figure 8).

The correlation matrix constructed for sensorimotor gating results (PPIs) in males revealed a positive correlation with object recognition data (DI) (Figure 8A). This effect was not observed in females (Figure 8B).

In males, PPI and DI showed positive correlations with the expression of the *GAD67* mRNA (*Gad1* gene), but they were negatively correlated with *Arc* expression. In addition, the expression of the *BRD3* mRNA exhibited a negative correlation with the results of the novel object recognition test. The level of the *BRD2* mRNA was proportional to the expression of the *c-Fos* and *Npas4* mRNAs, while *BRD3* expression was inversely proportional to the levels of these mRNAs. In contrast, *BRD3* expression was positively correlated with *Arc* expression. Moreover, *c-Fos* expression was positively correlated with *Npas4* expression, while *Arc* levels exhibited a negative correlation with the *GAD67* mRNA (Figure 8A). Thus, deficits in recognition memory and sensorimotor gating in males appear to be related to *GAD67* and *Arc* expression. Moreover, recognition memory formation appears to be correlated with *BRD3* and immediate early genes’ (*c-Fos*, *Npas4*, *Arc*) transcription activity.

In females, PPI negatively correlated with immediate early gene expression (*c-Fos*, *Npas4*, and *Arc*), and DI was negatively correlated with the level of the *BRD4* mRNA. However, *BRD4* expression exhibited a positive correlation with the *BRD3* mRNA level. In addition, *c-Fos* expression was positively correlated with *Npas4* and *Arc* gene transcription (Figure 8B). Thus, a deficit in sensorimotor gating in females appears to be related to immediate early gene expression (*c-Fos*, *Npas4,* and *Arc*), while recognition memory formation is rather correlated with transcription of *BRD* genes (*BRD4*, *BRD3*).

### 2.4. Proteomic Analysis of the Adult Medial Prefrontal Cortex

A proteomic study was performed to determine whether adolescent BET protein inhibition altered the protein landscape in the adult mPFC.

#### 2.4.1. Proteomic Analysis of Protein Expression in Males

iTRAQ identified 313 proteins and 1049 unique peptides. Among them, 48 proteins were identified as differentially expressed (Table 1). 

**Figure 9:**
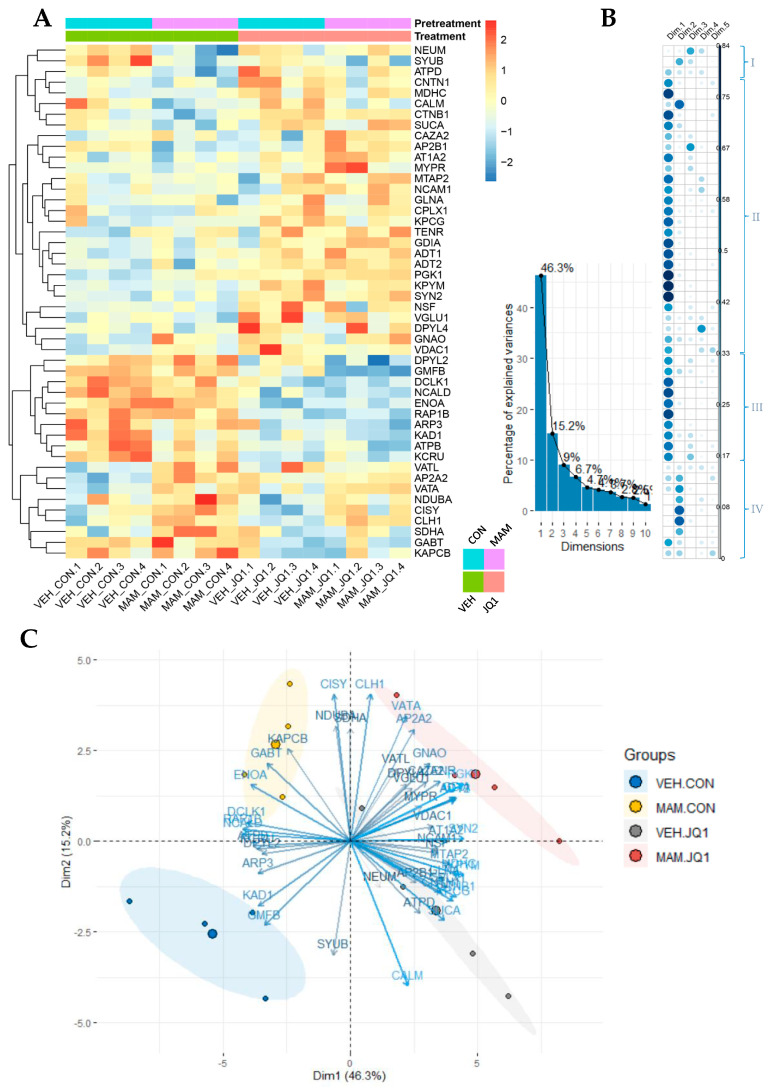
Proteomics study in the adult medial prefrontal cortex of males in the MAM-E17 model of schizophrenia. The rats were exposed to JQ1 or vehicle (CON) in early adolescence (P23–P29) and the analyses were performed in rats at P110. (**A**) Heatmap view of 48 differentially expressed proteins across groups (*p* < 0.050) identified from iTRAQ analysis. The colour scale illustrates the relative expression level of each protein across the 4 samples (biological replicates of mPFC) of each group: VEH-CON (1–4), MAM-CON (1–4), VEH-JQ1 (1–4), and MAM-JQ1 (1–4). Red and blue indicate higher and lower expression than the median expression value, respectively. The colour palette (right bar) represents standardised protein expression values (from red: upregulated, to blue: downregulated proteins). (**B**) Correlogram of the representation of the differentially expressed proteins depicted in the heatmap as well as their contribution to the principal components (Dim1–Dim5). Protein clusters (I–IV) on the right correspond to proteins visualised on the heatmap and correlogram. (**C**) PC biplot of the differentially expressed proteins. On the whole, the first component is linked to clusters affected by adolescent JQ1 treatment (cluster II and III). The second component corresponds to clusters affected by prenatal MAM administration (cluster I and IV). The first two principal components enabled us to visualise proteins specific for each treatment as well as the interaction between MAM and JQ1 administration. Abbreviated protein names are defined in Table 1.

The lowest Mascot score of the identified proteins was 35, with molecular masses ranging from 2 to 532 kDa (median 48 kDa). The heatmap in Figure 9A shows the protein expression levels in the analysed groups and a functional clustering analysis of the identified proteins. Proteins with similar expression patterns have relatively shorter Euclidean distances. Hierarchical clustering analysis with the Euclidean distance of proteins was used to identify patterns of protein expression following prenatal and postnatal treatment. Proteins were classified into 4 categories: 3 proteins (NEUM, SYUB, and ATPD) were affected by MAM treatment and their levels decreased mainly in the MAM-CON group (I); 26 proteins (CNTN1, MDHC, CALM, CTNB1, SUCA, CAZA2, AP2B1, AT1A2, MYPR, MTAP2, NCAM1, GLNA, CPLX1, KPCG, TENR, GDIA, ADT1, ADT2, PGK1, KPYM, SYN2, NSF, VGLU1, DPYL4, GNAO, and VDAC1) were altered by JQ1 treatment and their levels were increased in the VEH-JQ1 and MAM-JQ1 groups (II); 10 proteins (DPYL2, GMFB, DCLK1, NCALD, ENOA, RAP1B, ARP3, KAD1, ATPB, and KCRU) were also modulated by JQ1 treatment and their levels were decreased in the VEH-JQ1 and MAM-JQ1 groups (III); 9 proteins (VATL, AP2A2, VATA, NDUBA, CISY, CLH1, SDHA, GABT, and KAPCB) were altered by MAM and their levels were mainly increased in the MAM-CON group (IV).

- Principal component analysis (PCA)

PCA was conducted to identify unique components related to the interaction between both treatments: prenatal (MAM) and adolescent (JQ1). The first three components (Dim1, Dim2, Dim3) defined 70% of the total variance in our experiment (Figure 9B). An examination of the scatter plots of the proteins’ principal components revealed that the first component was linked to clusters II and III from the previous classification, namely, proteins regulated by adolescent JQ1 treatment (Figure 9B). The second component, reflecting reciprocally affected clusters I and IV from the previous analysis, identified proteins affected by prenatal MAM administration (Figure 9B). The third component corresponded to proteins classified in different defined clusters from a previous analysis; however, it comprised proteins functionally related to synapse and cytoskeleton organisation (AP2B1, CAZA2, DPYL2, MYPR, NEUM, SYUB, and VGLU1) or energy transfer processes (AT1A2, ATPB, KCRU, and VATL) (Figure 9B).

The two first components (Dim1, Dim2) differentiate all experimental groups (Figure 9C).

The proteomic analysis in the male adult mPFC identified distinctive molecular signatures related to either prenatal factor (MAM administration) or postnatal factor (adolescent JQ1 treatment) action.

#### 2.4.2. Proteomic Analysis of Protein Expression in Females

iTRAQ identified 348 proteins and 1190 unique peptides. Among them, 26 proteins were identified as differentially expressed (Table 2). 

**Figure 10:**
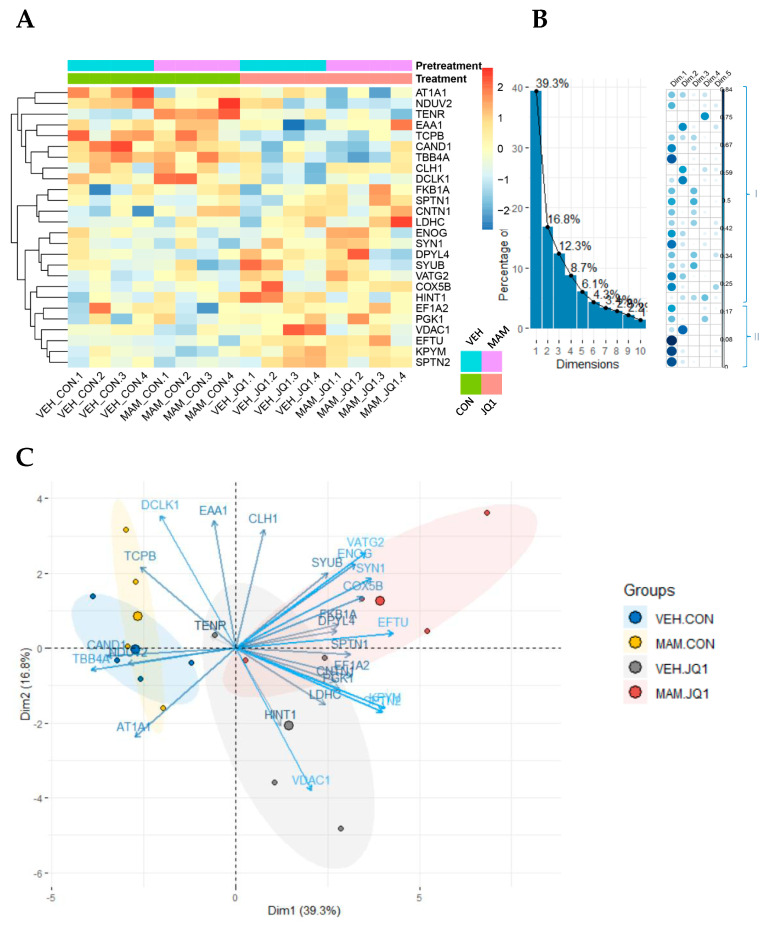
Proteomics study in the adult medial prefrontal cortex of females in the MAM-E17 model of schizophrenia. The rats were exposed to JQ1 or vehicle (CON) in early adolescence (P23–P29), and the analyses were performed in rats at P110. (**A**) Heatmap view of 26 differentially expressed proteins across groups (*p* < 0.050) identified from iTRAQ analysis. The colour scale illustrates the relative expression level of each protein across the 4 samples (biological replicates of mPFC) of each group: VEH-CON (1–4), MAM-CON (1–4), VEH-JQ1 (1–4), and MAM-JQ1 (1–4). Red and blue indicate higher and lower expression than the median expression value, respectively. The colour palette (right bar) represents standardised protein expression values (from red: upregulated, to blue: downregulated proteins). (**B**) Correlogram of the representation of the differentially expressed proteins depicted in the heatmap as well as their contribution to the principal components (Dim1–Dim5). Protein clusters (I–II) on the right correspond to proteins visualised on the heatmap and correlogram. (**C**) PC biplot of the differentially expressed proteins. On the whole, both components are linked to clusters (I and II) affected by adolescent JQ1 treatment. The first two principal components enabled us to visualise proteins engaged in JQ1 treatment. Abbreviated protein names are defined in Table 2.

The lowest Mascot score of the identified proteins was 26, with molecular masses ranging from 2 to 532 kDa (median 48 kDa). The heatmap in Figure 10A shows the protein expression levels in the analysed groups and a functional clustering analysis of the identified proteins. Proteins with similar expression patterns have relatively shorter Euclidean distances. Hierarchical clustering analysis with the Euclidean distance of proteins was used to identify patterns of protein expression following prenatal and postnatal treatment. Proteins were classified into 2 categories based on the effects of the JQ1 treatment: the levels of 9 proteins (AT1A1, NDUV2, TENR, EAA1, TCPB, CAND1, TBB4A, CLH1, and DCLK1) decreased in both the VEH-JQ1 and MAM-JQ1 groups (I), and the levels of 17 proteins (FKB1A, SPTN1, CNTN1, LDHC, ENOG, SYN1, DPYL4, SYUB, VATG2, COX5B, HINT1, EF1A2, PGK1, VDAC1, EFTU, KPYM, and SPTN2) increased in the VEH-JQ1 and MAM-JQ1 groups (II).

- Principal component analysis (PCA)

PCA was used to identify unique components related to the interaction between both treatments: prenatal MAM and adolescent JQ1. The first three components (Dim1, Dim2, Dim3) defined 70% of the total variance in our experiment (Figure 10B). An examination of the scatter plots of the proteins’ principal components revealed that the first component (Dim1) was linked to clusters I and II from the previous classification, indicating proteins regulated by adolescent JQ1 treatment (Figure 10B). Similar to Dim1, the second component, reflecting reciprocally affected clusters I and II (synaptic proteins: CLH1, DCLK1, and EAA1) from the previous analysis, identified proteins that were altered by adolescent JQ1 administration (Figure 10B). The third component corresponded to proteins classified in cluster I in the previous analysis (Figure 10B). The two first components (Dim1, Dim2) separate JQ1 groups from CON groups (Figure 10C).

Thus, proteomic analysis showed that changes in protein level in the female adult mPFC were related only to postnatal factor (adolescent JQ1 treatment).

## 3. Discussion

As shown in the present study, the administration of JQ1, an inhibitor of BET family proteins, in a defined period of adolescence affected behavioural responses and molecular and proteomic landscapes in the adult mPFC. The effects of JQ1 action were observed in animals of both sexes prenatally treated with VEH and MAM.

### 3.1. Behavioural Response

Behavioural tests were performed to analyse deficits in sensorimotor gating and recognition memory that are commonly observed in the MAM-E17 rat model of schizophrenia [30,31,32,33]. These behavioural impairments are considered models of the cognitive symptoms of schizophrenia. Sensorimotor gating is a pre-attentive process that is measured by PPI, and abnormalities in pre-attentive information processing may predict or lead to complex cognitive deficits [19]. PPI deficits were originally identified in patients with schizophrenia, and in preclinical studies, PPI was also used to investigate sensorimotor gating deficits in animal models of schizophrenia [19,20]. The memory impairment was examined using the novel object recognition test, which is known to have translational relevance to the domain of visual learning and memory [21,22], among the cognitive deficits observed in patients with schizophrenia [34]. The aforementioned task is a useful tool to evaluate recognition memory in animal models; however, some limitations related to the preference of a novel object should be considered, and the mechanisms involved in visual recognition memory of humans and rodents are not identical [21].

In the present study, we observed impairments in sensorimotor gating and memory recognition in male but not female MAM-E17 rats. Our previous studies also showed a decrease in PPI and memory impairments in the novel object recognition test in MAM-treated male rats [17,18,35]. However, in these studies, we did not analyse female rats. Literature data indicate some deficits in sensorimotor gating [36] or a reduction in discrimination index in novel object recognition in MAM-E17 females [29]. However, these studies were performed in Sprague-Dawley rats rather than in the Wistar Han rats used in our study. On the other hand, some clinical studies reported that healthy women showed lower levels of PPI than men [37,38], and a similar effect was reported in our study, where the PPI of males was higher than that of females. Moreover, the results from patients with schizophrenia are inconsistent, showing a decrease or unaffected sensorimotor gating in females [37,39], similar to those noted in the MAM-E17 model. Thus, female rats might not display all of the schizophrenia-like behavioural deficits induced by prenatal MAM treatment that were observed in male rats.

Adolescent JQ1 treatment affected the analysed behavioural parameters. In males, JQ1 impaired either memory recognition or sensorimotor gating in control rats (VEH-JQ1 group) but did not alter these parameters in the MAM group. In females, adolescent BET protein inhibition induced a reduction in novel object recognition in control rats; however, prenatal MAM treatment prevented this effect. On the other hand, JQ1 did not alter PPI but increased the startle amplitude in the VEH- and MAM-treated groups of female rats. To the best of our knowledge, the effect of JQ1 treatment has not yet been studied in acoustic startle response tests; thus, we are unable to easily compare our data with others. However, the role of BET proteins was determined in a novel object recognition test, and JQ1-treated adult animals exhibited impaired memory formation [12] and the blockade of an enhancement of recognition of a new object induced by an HDAC3 inhibitor [40]. Thus, our results add new knowledge of the role of BET proteins in the development of some behavioural responses, such as sensorimotor gating and recognition memory. Moreover, our data imply that the inhibition of BET family function during adolescence might disrupt these processes in adulthood, especially in males.

### 3.2. LTP in the Adult mPFC

The prefrontal cortex functionally and structurally transforms during the transition from adolescence to adulthood, which makes it very vulnerable to any environmental changes. Abnormal prefrontal cortical development might affect cognitive function in adulthood [41]. Thus, we analysed LTP formation in the adult mPFC to examine cortical cellular function, and we observed deficits in LTP only in the MAM-E17 male but not in the female mPFC. Our results are consistent with previous findings [27], showing a decrease in LTP only in the mPFC of MAM-treated adult male, not female mice. Thus, the available data suggest that male mPFC development might be more vulnerable to prenatal MAM treatment than female mPFC development.

Adolescent JQ1 administration only exerted a slight insignificant effect on LTP formation in the mPFC of control adult animals of both sexes and did not affect the MAM groups. On the other hand, JQ1 might significantly increase LTP in mouse hippocampal slices [42]. Thus, the role of the BET protein in LTP formation might depend on the brain region and neurodevelopmental period, and our results did not indicate sex differences in the effect of JQ1 on LTP in the mPFC.

### 3.3. Molecular Changes in the Adult mPFC

We examined the effect of adolescent functional blockade of BET proteins on *BRD* gene expression in the adult mPFC. Prenatal MAM administration decreased *BRD4* expression only in male rats, and it had no effect on *BRD2* and *BRD3* mRNA levels in either sex. To the best of our knowledge, this report is the first to show changes in *BRD* expression in the mPFC, which might suggest the involvement of the BET family in prefrontal cortical maturation and the development of schizophrenia-like abnormalities in male MAM-E17 rats. Adolescent JQ1 treatment did not affect *BRD* expression; however, it abolished the MAM-induced decrease in *BRD4* mRNA levels in the male mPFC. Thus, JQ1 administration during adolescence might normalise BRD cortical dysfunction.

In the present study, levels of IEGs (*c-Fos*, *Arc*, and *Npas4*) were examined as indirect markers of neuronal activity related to mPFC function. c-Fos and Npas4 are transcription factors that regulate downstream gene expression, and Arc encodes an effector protein that directly modulates cellular function [43]. c-Fos is expressed in an activity-dependent manner and is involved in complex learning and memory processes [23]. Arc is known to encode a synaptic protein that is involved in the generation of new synapses, and maintenance of the old synapses requires plasticity mechanisms (i.e., LTP) [23]. Npas4 plays an important role in adolescent maturation of the prefrontal inhibitory network [44] and regulates the excitatory and inhibitory balance at synaptic connections [45]. Thus, these IEGs are important for synaptic and neuronal plasticity, and they are considered involved in the development of psychiatric disorders (i.e., schizophrenia and autism) [23,46]. Prenatal MAM treatment did not alter the expression of any of the analysed IEGs in either sex. However, a gene-specific effect of adolescent JQ1 administration was observed. The effect of JQ1 on *c-Fos* expression was sex-specific, as it decreased the *c-Fos* mRNA level only in the male control and MAM groups. In contrast, adolescent inhibition of BET proteins induced an increase in *Arc* expression only in the MAM-treated group in both sexes. *Npas4* transcription was also altered by JQ1 treatment, and similar to the regulation of *c-Fos* mRNA expression, a reduction in the level of the *Npas4* mRNA was observed in male rats in the VEH and MAM groups. However, in females, a decrease in the Npas4 mRNA level was detected only in the control group. Nonetheless, correlation data showed a positive association between *c-Fos* and *Npas4* expression in both sexes that might suggest an important role of both genes in JQ1’s effect on neuronal activity in the mPFC. Recent evidence reported that JQ1 regulates *c-Fos* and *Arc* gene expression in vitro [12]. Thus, our results confirm previous findings related to c-Fos and Arc regulation, but also suggest that *Npas4* transcription might be regulated by JQ1. The direction of changes in *c-Fos* and *Npas4* expression observed in the present study were related to decreases in behavioural response (sensorimotor gating and novel object recognition) induced by JQ1 treatment in males. In females, only decreased levels of the *Npas4* transcript in the mPFC might be related to an effect of JQ1 on the behavioural response of the control group (reduction in DI in the novel object recognition task and lack of effect on sensorimotor gating), as well as some slight effect on LTP observed in the female cortex. Thus, adolescent JQ1 administration modified neuronal and synaptic activity in the adult mPFC by altering expression of IEGs, and males might be more vulnerable to adolescent JQ1 treatment than females.

Our previous study showed a decrease in the mRNA levels of both *GAD67* and *PV* induced by prenatal MAM treatment in the adult male mPFC [17,18,25]. In the present study, we also observed a decrease in *GAD67* and *PV* expression in the mPFC of males but not females. Adolescent JQ1 treatment did not affect the expression of either *GAD67* or *PV* in the mPFC of either sex. Thus, the dysfunction of GABAergic interneurons in the male mPFC might be involved in the deficits in sensorimotor gating, memory recognition, and LTP observed in MAM-treated males, but not female rats.

A correlation study of behavioural responses and gene expression also suggested sex-specific relationships in the association between these factors. Behavioural responses correlated with the expression of different genes in males (*Gad1*) and females (activity-dependent genes: *c-Fos* and *Npas4*); however, *Arc* expression appears to be important in both sexes. The correlations between the analysed genes were also different in males and females, and the correlations between the expression of *BRD* genes and *IEGs* were stronger in males than in females. The latter observation suggests that alterations in *BRD* gene transcription might exert a stronger effect on *IEG* gene expression in males than in females. Therefore, adolescent treatment with JQ1 might exert a stronger effect on developmental abnormalities in males than females.

### 3.4. Proteomic Alterations in the Adult mPFC

Our results revealed functional and molecular changes in the adult mPFC of both sexes induced by both MAM and JQ1 administration, but the main changes were detected in males. Thus, we performed a proteomic study to investigate proteins that might be affected by either prenatal or postnatal treatment in both sexes.

Administration of either the prenatal risk factor MAM or JQ1 during adolescence altered protein levels in the male mPFC, and based on the direction of changes, proteins were divided into 4 categories. The first category of proteins showed decreased expression in the MAM-CON group. Two are related to synapse function (NEUM and SYUB), and one is involved in energy transfer processes (ATPD). The second category is a large group of proteins showing increased levels in the JQ1 groups (VEH-JQ1 and MAM-JQ1). They are related to synapse function and signalling pathways (CNTN1, CALM, CTNB1, AP2B1, AT1A2, NCAM1, CPLX1, KPCG, TENR, SYN2, NSF, VGLU1, GNAO, and VDAC1), cytoskeletal organisation (CAZA2, MYPR, MTAP2, GDIA, and DPYL4), metabolic processes (GLNA, PGK1, and KPYM), and energy transfer processes (MDHC, SUCA, ADT1, and ADT2). The levels of proteins in the third category were decreased in the JQ1 groups (VEH-JQ1 and MAM-JQ1), and they are mainly involved in synapse function (DCKL1 and RAP1B), cytoskeletal organisation (DPYL2, GMFB, NCALD, and ARP3), and metabolic and energy transfer processes (ENOA, ATPB, and KCRU). Levels of proteins in the fourth category were increased mainly in the MAM-CON group, and they are related to synapse function and signalling processes (AP2A2, CLH1, GABT, and KAPCB) or metabolic and energy transfer processes (CISY, SDHA VATL, VATA, and NDUBA).

Proteins identified in the female mPFC were divided mainly into 2 categories based on the effects of the JQ1 treatment. Levels of proteins in the first category were decreased in both the VEH-JQ1 and MAM-JQ1 groups, and they are related to synapse function or cytoskeletal organisation (AT1A1, TENR, EAA1, CAND1, TBB4A, CLH1, and DCLK1) or are involved in metabolic and energy transfer processes (NDUV2 and TCPB). The levels of proteins in the second category increased in the VEH-JQ1 and MAM-JQ1 groups, and they are involved in synaptic (FKB1A, CNTN1, SYN1, and VDAC1) and cytoskeletal organisation (SPTN1, DPYL4, SYUB, and SPTN2), as well as metabolic (LDHC, ENOG, PGK1, KPYM, and HINT1) and energy transfer processes (EF1A, EFTU, COX5B, and VATG2).

Our previous proteomic study of the male adult mPFC showed a MAM-induced decrease in the levels of proteins related to synapse and cytoskeletal organisation and a MAM-evoked increase in the levels of proteins related to metabolic and energy transfer processes [47]. The present study also showed a decrease in the levels of synaptic proteins in MAM-treated males, which might suggest changes in neuropil reorganisation in the adult mPFC and might explain the reduction in the adult mPFC volume of MAM-E17 males reported in our previous study [18]. In female mPFC, the levels of proteins related to synapse function and signalling pathways, as well as metabolic and energetic processes, were not changed after MAM treatment. The proteomic analysis identified 85% more altered proteins in MAM- and JQ1-treated males than in females, where only 15% of proteins were altered. Thus, MAM administration mainly altered protein levels in the male mPFC. Sex differences in the proteomic landscape of the MAM-treated adult mPFC were also observed by other researchers [27]. A proteomic study performed in the human prefrontal cortex indicated alterations in proteins in patients with schizophrenia. These proteins are related to dysfunctional metabolism, cytoskeleton-related abnormalities, and cellular connections [48]. Thus, the proteins that were altered in our study might be involved in schizophrenia-like abnormalities observed mainly in male MAM-treated rats.

The robust effect of adolescent JQ1 treatment on protein levels in the mPFC of both VEH- and MAM-treated rats was observed in synaptic and cytoskeletal proteins, as well as proteins involved in metabolic and energy transfer processes, in both sexes. The levels of synaptic proteins were altered in both sexes (CLH1, CNTN1, DCLK1, SYUB, TENR, and VDAC1); however, the TENR protein was regulated in a sex-specific manner, and CLH1 and SYUB were regulated only in females by JQ1. Our results showing changes induced by JQ1 in synaptic proteins are consistent with RNA-sequencing data showing JQ1-induced alterations in the transcription of synaptic proteins and receptors in neurons [12].

## 4. Materials and Methods

### 4.1. Animals and Treatment

**Figure 11:**
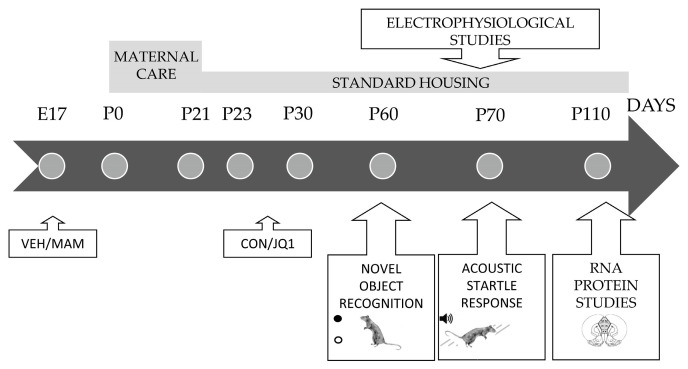
The schematic diagram depicts the adolescent administration of the inhibitor of BET proteins, JQ1 or vehicle (CON), of rats treated with vehicle (VEH) or MAM at embryonic day 17 (E17); P (postnatal days of life).

Pregnant dams (*Wistar Han rats*) were obtained from an animal provider (Charles River, Sulzfeld, Germany) when the foetuses were aged embryonic day 15 (E15) and housed individually in polycarbonate cages (26.5 × 18 × 42 cm). The rats were randomly assigned to experimental groups; at E17, the pregnant females were intraperitoneally (i.p.) injected with 22 mg/kg of methylazoxymethanol acetate (MAM, MRIGlobal, Kansas City, MO, USA) or saline vehicle (0.9% NaCl) [7,17,18,35]. The offspring were weaned 21 days after birth, and males and females were used in our experiments. The rats were housed in large polycarbonate boxes (38 × 20 × 60 cm) in groups of 4 in the same sex on an artificial 12/12 h light/dark cycle (lights on at 7 A.M.) with ad libitum access to food and water. The experimental groups consisted of animals chosen randomly from different litters (total 16) to avoid litter effects (maximum 2 rats from a single litter per group). The BET inhibitor (+)JQ1 ((6*S*)-4-(4-chlorophenyl)-2,3,9-trimethyl-6*H*-thieno[3,2-*f*][1,2,4]triazolo[4,3-*a*][1,4]diazepine-6-acetic acid 1,1-dimethylethyl ester; STI, Poznan, Poland) was administered at a dose of 25 mg/kg i.p. once a day for 7 consecutive days in early adolescence (23rd–29th days of life). JQ1 was dissolved in 10% DMSO and then diluted in corn oil. All experiments (behavioural and biochemical) were performed on four experimental groups (VEH-CON, VEH-JQ1, MAM-CON, and MAM-JQ1) using the same set of animals, but electrophysiological studies were performed using different sets of rats (Figure 11). The behavioural experiments were performed in adult rats as described below. Rats performed a novel object recognition test at P60 followed by an acoustic startle response test a week later. For the behavioural studies, each experimental group consisted of 12 rats per group. The animals were sacrificed a month after the last test, and brain tissue was stored until use in biochemical experiments. Biochemical studies (qRT-PCR and proteomics) were performed on the same mPFC samples obtained from adult rats at P110, and each experimental group consisted of 4–6 animals. A separate set of animals was used for the electrophysiological study performed on the mPFC of adult animals aged P60–P110, and each group consisted of 6–9 animals.

The study was carried out in strict accordance with the recommendations in the European Council Guide for the Care and Use of Laboratory Animals (86/609/EEC) and revised by Directive 2010/63/UE for the protection of animals used for scientific purposes. The protocols (permission number 138/2020; data of approval 4 June 2020) were approved by the Committee for Laboratory Animal Welfare and the Ethics of the Maj Institute of Pharmacology, Polish Academy of Sciences in Kraków, and were conducted in accordance with the current version of the Polish Law on the Protection of Animals (266/2015).

### 4.2. Behavioural Study

#### 4.2.1. Novel Object Recognition Test

The study was performed as described previously with some modifications [17,35]. Rats were tested for memory deficits in dimly lit (18 lx) cages made of black Plexiglas (60 × 60 × 30 cm). After each measurement, the floor and walls were cleaned and dried. Twenty-four hours before training, the animals were habituated to the arena (without any objects) for 10 min. Objects used for the discrimination task included a black glass cylinder and a white plastic cuboid filled with water. The objects were placed in two opposite corners of the arena, approximately 10 cm from the walls. The rats were placed in the middle of the arena, and during the training session (acquisition), two identical objects (A and A1) were presented for 5 min (half of the animals were presented with two black glass cylinders, and the other half with two white plastic cuboids). After a 1 h rest in the home cage, the rats were again placed in the same Plexiglas cage as in the previous test and presented with two objects for 3 min, but one of the objects was replaced by a novel object (A—familiar and B—novel) (recognition session). The objects were cleaned with a 70% ethanol solution between sessions. Exploration was defined as sniffing the object and/or touching it with the nose or forepaws. The animal behaviour was automatically recorded by cameras (SJ8Pro SJCAM, China) and next, videos were analysed using the Solomon Coder program (https://solomon.andraspeter.com, accessed on 5 August 2021). Animals showing less than 5 s of exploration time were excluded from the experiment. Based on the time spent exploring (E) each object, a discrimination index (DI) was calculated: DI = (E_B_ − E_A_)/(E_A_ + E_B_).

#### 4.2.2. Acoustic Startle Response Test

The efficacy of sensorimotor gating was measured by recording pre-pulse inhibition (PPI) of the acoustic startle response, and reactivity was measured using a startle apparatus (SR-LAB, San Diego Instruments, San Diego, CA, USA), as described previously [7,17,18,35]. Briefly, after a 5 min habituation period that included playing background white noise at 65 dB, two types of acoustic stimuli were presented in a random order: an acoustic stimulus alone ((P), duration 40 ms, intensity 120 dB) and an acoustic stimulus preceded by an acoustic pre-pulse ((PP), duration 20 ms, intensities: 70, 75, and 80) applied 80 ms before the stimulus (P). During each experimental session, 20 trials of each type were presented with randomised interstimulus intervals ranging from 5 to 20 s in length. The amplitudes were averaged separately for each animal and for both types of trials (stimulus alone, P; stimulus preceded by the pre-pulse, PP). The degree of pre-pulse inhibition was calculated as the percentage of inhibition (%PPI) using the following formula: [(P − PP)/P] × 100.

### 4.3. Electrophysiological Study

The electrophysiological procedure has been described previously [49]. The procedure was performed on adult rats between P60 and P110. The rats from each experimental group were tested in a counterbalanced, alternating order. Rats were anesthetised with isoflurane (Aerrane, Baxter, Warsaw, Poland) and decapitated. The brain was rapidly removed and immersed in an ice-cold artificial cerebrospinal fluid (ACSF) of the following composition (in mM): 130 NaCl, 5 KCl, 2.5 CaCl_2_, 1.3 MgSO_4_, 1.25 NaH_2_PO_4_, 26 NaHCO_3_, and 10 d-glucose, and ACSF was bubbled with a mixture of 95% O_2_ and 5% CO_2_ (pH 7.4). A block of tissue containing the mPFC was dissected and cut into 410 µm thick sections along the coronal plane using a vibratome at 1 °C (VT 1000S, Leica, Germany). The sections were transferred into a recording chamber with a fluid–gas interface and superfused at a rate of 2 mL/min with warm (32 ± 0.5 °C)-modified ACSF containing (in mM): 130 NaCl, 2 KCl, 2.5 CaCl_2_, 1.3 MgSO_4_, 1.25 NaH_2_PO_4_, 26 NaHCO_3_, and 10 D-glucose. The recordings started 2–4 h after the sections were prepared. The field potentials were evoked using square-wave stimuli (duration 0.2 ms) applied by a constant current isolation unit (WPI, Germany) at 0.016 Hz via a bipolar concentric Pt-Ir electrode (FHC, Bowdoin, ME, USA). The electrode was placed in layer II/III of the mPFC. A recording micropipette filled with ACSF (resistance of approximately 2–4 MΩ) was placed in cortical layer V of the mPFC. The responses were amplified (500×), filtered (0.1 Hz–1.3 kHz), and acquired at a 10 kHz sampling rate (EXT 10-C amplifier, NPI, Germany) using a Micro1401 interface and Signal 2 software (CED, Cambridge, UK).

After stabilisation of the responses, stimulus/response (input–output) curves were determined individually for each section by applying square stimuli of increasing intensities (duration of 0.2 ms; range of 0–50 µA, step 5 µA; range of 50–100 µA, step 10 µA; applied at 15 s intervals). Then, the stimulation intensity was adjusted to evoke a response corresponding to 40% of the maximum amplitude. When the signals became stable over a baseline period of 15 min, LTP was induced in the mPFC with a sequence of five stimulus bursts (15 s apart). A single burst consisted of 5 individual square pulses (duration of 0.3 ms, applied at 100 Hz) that were repeated ten times (0.2 s apart). The recording then continued for an additional 90 min. The response amplitude of the individual sections was normalised to the percent change relative to the baseline values. The amount of LTP was determined by comparing the data from the baseline recording averaged over the last 15 min to the data from the post-tetanic recording averaged over the last 15 min.

### 4.4. Biochemical Study

#### 4.4.1. Tissue Preparation

After the rats were decapitated, the brains were removed, cooled on ice, and sliced into 1 mm coronal sections using a rodent brain matrix (Ted Pella, Inc., Redding, CA, USA). The mPFC was dissected from coronal sections, frozen in liquid nitrogen, and stored at −20 °C. Simultaneous extraction of proteins and nucleic acids from the same experimental sample was performed using commercially available kits (Nucleospin RNA/Protein, Macherey-Nagel, RNAeasy, Qiagen, Wroclaw, Poland) and columns (Amicon Ultra, Millipore, Warsaw, Poland) with some modifications. RNA was extracted using the RNeasy kit according to the manufacturer’s instructions (www1.qiagen.com/literature/handbooks, accessed on 30 June 2021). The flow through was collected, and proteins were extracted using a Nucleospin RNA/Protein kit according to the manufacturer’s instruction (www.mn-net.com/media/pdf, accessed on 30 June 2021) and purified with Amicon Ultra Centrifugal Filter Units (Amicon). Proteins were dissolved in Dissociation Buffer (SCIEX) and quantified using the bicinchoninic acid (BCA) Protein Assay kit (Pierce, Rockford, IL, USA) according to the manufacturer’s instructions (www.piercenet.com/files/1296dh4.pdf, accessed on 30 June 2021). 

The obtained mRNA and proteins were used for qRT-PCR or proteomic studies, respectively.

#### 4.4.2. qRT-PCR Study

qRT-PCR was performed using the QuantStudio 12K Flex Real-Time PCR System (Applied Biosystems, Warsaw, Poland) as previously described [25]. The gene-specific primers and probes (see details in Table 3) from the TaqMan Gene Expression Assays (Applied Biosystems) were used, and 1 μL of each sample was used for cDNA amplification. Amplification was performed using the TaqMan Universal PCR Master Mix (Applied Biosystems) under the following conditions: 50 °C for 2 min and 95 °C for 10 min, followed by 40 cycles of 95 °C for 15 s and 60 °C for 1 min. The expression of the glyceraldehyde-3-phosphate dehydrogenase (Gapdh) transcript (assay Rn99999916_s1) was quantified to control for variations in cDNA levels. All qRT-PCR experiments were performed in duplicate and included no-template controls. The cycle threshold values were calculated automatically. Relative quantification was performed using the comparative threshold (CT) method, 2−ΔCT, where ΔCT = (CT, target gene − CT, reference gene).

#### 4.4.3. Proteomic Analysis

- Protein Extraction

The study was performed as described previously with some modifications [47]. Protein concentrations of the obtained fraction were determined using a BCA protein assay (Sigma-Aldrich, Poznan, Poland). One hundred micrograms of protein from each sample were used for analysis. Afterwards, samples were reduced by Tris-(2-carboxyethyl)-phosphine (TCEP, Sigma-Aldrich, and cysteines were blocked according to the iTRAQ protocol (AB Sciex, Framingham, MA, USA). Proteins were digested using Sequencing Grade Modified Trypsin (from Promega, Warsaw, Poland) overnight at 37 °C.

- Stable isotope labelling

Samples were labelled using iTRAQ reagents (AB Sciex, Framingham, MA, USA), as recommended by the manufacturer for 8-plex iTRAQ. Labelled samples were combined and dried in a vacuum centrifuge (Labconco, VWR, Gdansk, Poland).

- Strong cation-exchange fractionation

Lyophilisates were dissolved in 10 mM of potassium phosphate containing 25% acetonitrile at pH 3.0. The samples were purified on an Applied Biosystems Cation-Exchange Cartridge. The peptides were eluted from the column with 10 mM of potassium phosphate containing 25% acetonitrile at pH 3.0 and 350 mM of potassium chloride (Sigma-Aldrich). The eluate was dried in a vacuum centrifuge, reconstituted in 2% acetonitrile and 0.1% formic acid, and subjected to MacroSpin Columns with C18 resin (the Nest Group, Inc., Ipswich, MA, USA) to remove salts. The samples were eluted with 80% acetonitrile containing 0.1% formic acid, dried in a vacuum centrifuge again, and redissolved in 2% acetonitrile containing 0.1% formic acid for nano-LC-MALDI analyses.

- Chromatographic separation and spotting

Chromatographic separation of peptides was achieved on a Proxeon EASY-nLC system (Bruker Daltonics, Bremen, Germany) equipped with a 2 cm long, 100 µm ID precolumn containing 5 µm BioSphere C18 resin (NanoSeparations, Nieuwkoop, The Netherlands) and a 10 cm long, 75 µm ID fused silica column containing 3 µm BioSphere C18 resin (NanoSeparations, The Netherlands). The gradient was formed using 0.1% formic acid in water (solvent A) and 0.1% formic acid in acetonitrile (solvent B). The system was controlled by Hystar software (Bruker Daltonics). A gradient was established from 2% to 35% B in 50 min, that increased to 90% B at 55 min and then was maintained until 65 min. Samples were loaded from a cooled (7 °C) autosampler and separated with a linear gradient that was formed at a flow rate of 300 nl/min. Chromatographic separation was controlled by the Hystar system (Bruker Daltonics). The LC eluent was directly mixed with α-cyano-4-hydroxycinnamic acid matrix (HCCA, Bruker Daltonics) and deposited on an MTP AnchorChip 1536 BC plate (Bruker, Daltonik). The LC eluent was spotted with a Proteineer fc II (Bruker, Daltonik). Spots were deposited every 15 s, and 192 fractions were spotted in total for one sample.

- LC-MALDI-TOF/TOF analysis

All data were acquired on an ultrafleXtreme (BrukerDaltonik, Bremen, Germany) mass spectrometer with a TOF/TOF analyser. All MS spectra were recorded in positive reflector mode (700–3500 Da). For MS data, 4000 shots were accumulated for each spectrum. Collected data were analysed using WARP-LC software (Bruker Daltonics, Bremen, Germany). Search parameters were set as follows: taxonomy: Rodentia (UniProt/Swiss-Prot, Expasy, Swiss Institute of Bioinformatics (www.expasy.org, accessed on 11 August 2021); enzyme: trypsin; missed cleavage sites allowed: 1; fixed modification: methylation (C); variable modifications: methionine oxidation (M), iTRAQ8plex (K), iTRAQ8plex (N-term), and iTRAQ8plex (Y); parent mass error: 50 ppm; peptide fragment mass tolerance: 0.6 Da.

- Protein interaction network analysis

Differentially expressed proteins identified in the present study were subjected to a pathway analysis. For this purpose, the Swiss-Prot accession numbers were inserted into STRING (Search Tool for the Retrieval of Interacting Genes/Proteins) software (http://string.embl.de, accessed on 15 June 2021).

- Database searching

In addition, all the identified proteins were searched using UniProtKB and the Panther Database to establish their molecular functions and interactions.

- Proteomic data depository

The mass spectrometry proteomics data have been deposited to the ProteomeXchange Consortium via the PRIDE partner repository with the dataset identifiers PXD027050 and 10.6019/PXD027050.

### 4.5. Statistical Analysis

The results are presented as the group means ± the standard errors of the means (SEM). Statistical evaluations were performed using Statistica software and consisted of one-way or two-way repeated measures analyses of variance (ANOVAs), as indicated in the figures, followed by the Tukey post hoc test. The between- (prenatal and postnatal treatment) and within-subjects (pre-pulse intensity for the acoustic startle response test or objects for the novel object recognition test) factors were used for the ANOVAs. Differences were considered significant at *p* < 0.05.

All statistical analyses of proteomics data were performed using R software version 3.6.2. Corrections for multiple testing were performed using Bonferroni’s method. Proteomic data were subjected to two-way ANOVA with prenatal or postnatal treatment as factors, followed by correction for multiple testing using Bonferroni’s method. Differences were considered significant at *p* < 0.05.

Associations among gene expression and proteomic and behavioural data were estimated by calculating Pearson’s rank-based correlation coefficients, followed by correction for multiple testing using the Benjamini–Hochberg method to decrease the false discovery rate.

## 5. Conclusions

In our study, a prenatal MAM treatment exerted a sex-specific effect on the development of schizophrenia-like abnormalities, and only males were affected. Adolescent administration of JQ1 altered behavioural responses in control animals, mainly in males, but not in rats of both sexes in MAM-treated groups. Deficits in LTP in the mPFC were only observed in male MAM-treated animals, and adolescent JQ1 treatment did not affect cellular memory in either sex. The results from the electrophysiological study are consistent with proteomic data showing changes in synaptic proteins only in male MAM-treated rats. On the other hand, protein alterations induced by JQ1 did not affect cortical LTP. Thus, prenatal MAM treatment might change the responsiveness to BET protein functions during adolescence.

JQ1 treatment during adolescence might affect the direction of prefrontal cortical development in both sexes by modulating gene expression and the protein landscape. Although there are some limitations, and caution is required in comparison animal and human studies, the aforementioned results might be important not only for determining the emergence of schizophrenia but also for the administration of JQ1 therapy to this age group, which might be practised in children who are oncology patients [50].

## Figures and Tables

**Table 1:** The list of 48 proteins identified in the adult medial prefrontal cortex of the male rats in the proteomic analysis. For each protein match, Mascot calculates an overall Protein Score. This number reflects the combined scores of all observed mass spectra that can be matched to amino acid sequences within that protein. A higher score indicates a more confident match.

Accession	Gene	Protein	MW.kDa	Scores	Peptides
ADT1	Slc25a4	ADP/ATP translocase 1	33	139.5	5
ADT2	Slc25a5	ADP/ATP translocase 2	32.9	96.9	4
AP2A2	Ap2a2	AP-2 complex subunit alpha-2	104	293.2	4
AP2B1	Ap2b1	AP-2 complex subunit beta	104.5	109.5	3
ARP3	Actr3	Actin-related protein 3	47.3	46.6	1
AT1A2	Atp1a2	Sodium/potassium-transporting ATPase subunit alpha-2	112.1	220.5	3
ATPB	Atp5f1b	ATP synthase subunit beta, mitochondrial	56.3	748.1	12
ATPD	Atp5d	ATP synthase subunit delta	17.6	54.6	2
CALM	Calm1	Calmodulin	16.8	98.8	2
CAZA2	Capza2	F-actin-capping protein subunit alpha-2	32.9	68.5	1
CISY	Cs	Citrate synthase	23.9	59.6	1
CLH1	Cltc	Clathrin heavy chain 1	191.4	671	13
CNTN1	Cntn1	Contactin-1	113.4	158.2	4
CPLX1	Cplx1	Complexin-1	15.1	41.4	1
CTNB1	Ctnnb1	Catenin beta-1	85.4	75.3	1
DCLK1	Dclk1	Serine/threonine-protein kinase DCLK1	84.1	51.8	1
DPYL2	Dpysl2	Dihydropyrimidinase-related protein 2	62.2	618.8	8
DPYL4	Dpysl4	Dihydropyrimidinase-related protein 4	61	62.6	2
ENOA	Eno1	Alpha-enolase	23.8	123.7	2
GABT	Abat	4-aminobutyrate aminotransferase	56.4	80.5	2
GDIA	Gdi1	Rab GDP dissociation inhibitor alpha	50.5	164	3
GLNA	Glul	Glutamine synthetase	42.1	148.5	2
GMFB	Gmfb	Glia maturation factor beta	16.7	73.1	1
GNAO	Gnao1	Guanine nucleotide-binding protein G(o) subunit alpha	40	198	3
KAD1	Ak1	Adenylate kinase isoenzyme 1	21.6	75.5	1
KAPCB	Prkacb	cAMP-dependent protein kinase catalytic subunit beta	40.7	39.5	1
KCRU	Ckmt1	Creatine kinase U-type, mitochondrial	47	112.7	2
KPCG	Prkcg	Protein kinase C gamma	78.3	40.8	1
KPYM	Pkm	Pyruvate kinase PKM	57.8	440.6	9
MDHC	Mdh1	Malate dehydrogenase	36.5	48.5	2
MTAP2	Map2	Microtubule-associated protein 2	199	112.8	1
MYPR	Plp1	Myelin proteolipid protein	30.1	54.7	1
NCALD	Ncald	Neurocalcin-delta	22.2	46.4	1
NCAM1	Ncam1	Neural cell adhesion molecule 1	94.6	79.6	2
NDUBA	Ndufb10	NADH dehydrogenase [ubiquinone] 1 beta subcomplex subunit 10	21	45.9	1
NEUM	Gap43	Neuromodulin	23.6	132.3	4
NSF	Nsf	Vesicle-fusing ATPase	82.5	144.4	5
PGK1	Pgk1	Phosphoglycerate kinase 1	44.5	187	4
RAP1B	Rap1b	Ras-related protein Rap-1b	20.8	73.2	1
SDHA	Sdha	Succinate dehydrogenase [ubiquinone] flavoprotein subunit, mitochondrial	60	68.5	1
SUCA	Suclg1	Succinyl-CoA ligase [ADP/GDP-forming] subunit alpha, mitochondrial	36.1	74.3	1
SYN2	Syn2	Synapsin-2	63.3	114.9	3
SYUB	Sncb	Beta-synuclein	14	47.4	1
TENR	Tnr	Tenascin-R	149.5	124.8	2
VATA	Atp6v1a	V-type proton ATPase catalytic subunit A	68.3	357.9	4
VATL	Atp6v0c	V-type proton ATPase 16 kDa proteolipid subunit	15.8	40.9	1
VDAC1	Vdac1	Voltage-dependent anion-selective channel protein 1	32.3	504.4	7
VGLU1	Slc17a7	Vesicular glutamate transporter 1	61.6	127.5	2

**Table 2:** The list of 26 proteins identified in the adult medial prefrontal cortex of the female rats in the proteomic analysis. For each protein match, Mascot calculates an overall Protein Score. This number reflects the combined scores of all observed mass spectra that can be matched to amino acid sequences within that protein. A higher score indicates a more confident match.

Accession	Gene	Protein	MW.kDa	Scores	Peptides
AT1A1	Atp1a1	Sodium/potassium-transporting ATPase subunit alpha-1	112.9	439.5	6
CAND1	Cand1	Cullin-associated NEDD8-dissociated protein 1	136.2	176	3
CLH1	Cltc	Clathrin heavy chain 1	191.4	703.3	12
CNTN1	Cntn1	Contactin-1	113.4	88.6	2
COX5B	Cox5b	Cytochrome c oxidase subunit 5B, mitochondrial	13.9	51.7	2
DCLK1	Dclk1	Serine/threonine-protein kinase DCLK1	84.1	61.3	1
DPYL4	Dpysl4	Dihydropyrimidinase-related protein 4	61	111.7	2
EAA1	Slc1a3	Excitatory amino acid transporter 1	59.6	69	1
EF1A2	Eef1a2	Elongation factor 1-alpha 2	50.4	153.5	4
EFTU	Tufm	Elongation factor Tu, mitochondrial	18.8	54.4	1
ENOG	Eno2	Gamma-enolase	47.1	263	5
FKB1A	Fkbp1a	Peptidyl-prolyl cis-trans isomerase FKBP1A	11.9	38.8	1
HINT1	Hint1	Histidine triad nucleotide-binding protein 1	13.8	79.1	1
KPYM	Pkm	Pyruvate kinase PKM	57.8	467.5	10
LDHC	Ldhc	L-lactate dehydrogenase C chain	35.7	53	1
NDUV2	Ndufv2	NADH dehydrogenase [ubiquinone] flavoprotein 2, mitochondria	27.4	69.4	1
PGK1	Pgk1	Phosphoglycerate kinase 1	44.5	213.2	4
SPTN1	Sptan1	Spectrin alpha chain, non-erythrocytic 1	284.5	951.5	18
SPTN2	Sptbn2	Spectrin beta chain, non-erythrocytic 2	270.9	100.4	2
SYN1	Syn1	Synapsin-1	73.9	284.5	6
SYUB	Sncb	Beta-synuclein	14	65.4	2
TBB4A	Tubb4a	Tubulin beta-4A chain	49.6	930.5	20
TCPB	Cct2	T-complex protein 1 subunit beta (Fragments)	15.9	70.5	1
TENR	Tnr	Tenascin-R	149.5	137.3	2
VATG2	Atp6v1g2	V-type proton ATPase subunit G 2	13.6	36.6	1
VDAC1	Vdac1	Voltage-dependent anion-selective channel protein 1	30.7	546.4	7

**Table 3:** A list of a gene-specific primers and probes.

Assay ID	Gene Symbol	Gene Name
Rn99999916_s1	GAPDH	glyceraldehyde-3-phosphate dehydrogenase
Rn01435739_m1	BRD3	bromodomain containing 3
Rn01428703_m1	BRD2	bromodomain containing 2
Rn01535560_m1	BRD4	bromodomain containing 4
Rn00574541_m1	Pvalb	parvalbumin
Rn00566593_m1	Gad1	glutamate decarboxylase 1
Rn02396759_m1	Fos	FBJosteosarcoma oncogene
Rn01454622_g1	Npas4	neuronal PASdomain protein 4
Rn00571208_g1	Arc	Activity-regulated cytoskeletal

## Data Availability

The mass spectrometry proteomics data have been deposited to the ProteomeXchange Consortium via the PRIDE partner repository with the dataset identifiers PXD027050 and 10.6019/PXD027050.
